# Real-world evaluation of belumosudil for chronic ocular graft-versus-host-disease: Efficacy and outcomes

**DOI:** 10.1038/s41409-026-02846-9

**Published:** 2026-03-23

**Authors:** Leonie Menghesha, Tim Richardson, Jana Knuever, Udo Holtick, Christof Scheid, Michael E. Stern, Philipp Steven

**Affiliations:** 1 Competence Center for Ocular GVHD, Clinic I of Internal Medicine and Department of Ophthalmology, University of Cologne, Faculty of Medicine and University Hospital Cologne, Cologne, Germany; 2 Clinic I of Internal Medicine, Center of Integrated Oncology Köln Bonn, University of Cologne, Faculty of Medicine and University Hospital Cologne, Cologne, Germany; 3 Centre for Skin Diseases, Department of Dermatology and Allergy, University Hospital Bonn, Bonn, Germany

**Keywords:** Haematological cancer, Haematological cancer

## Introduction

Graft-versus-host disease (GVHD) affects up to 50% of patients after allogeneic hematopoietic stem cell transplantation (aHCT) [1–3]. Chronic ocular GVHD (oGVHD) is particularly debilitating, characterized by severe ocular surface disease, inflammation and fibrosis of lacrimal gland, cornea and conjunctiva. Current treatment options for chronic oGVHD are limited, relying on topical therapies, which may not provide significant improvement in advanced cases. Unfortunately, once the window of opportunity for early treatment initiation is missed, the disease often tends to progress. Belumosudil (BEL), a selective Rho-associated coiled-coil containing protein kinase 2 (ROCK2) inhibitor, has emerged as a promising therapeutic oral agent for cGVHD. By modulating fibrotic pathways and inflammatory responses, BEL has demonstrated clinical efficacy in reducing systemic cGVHD manifestations in therapy-refractory and severe cases [4]. However, its effect on the eyes has not been characterized in detail [5–7]. Therefore, this study aims to evaluate the effect of BEL on chronic oGVHD. By exploring the potential to address a critical unmet need in oGVHD treatment, we hope to expand the therapeutic landscape for this challenging condition and improve oGVHD management.

## Methods

This single-centre retrospective study is based on data acquired at the Departments of Ophthalmology and Internal Medicine, University Hospital of Cologne, Germany between June 2024 and April 2025. All tenets of the declaration of Helsinki have been followed.

Ocular GVHD severity was evaluated using the National Institutes of Health (NIH) diagnostic criteria and the international chronic oGVHD diagnostic criteria (ICCGVHD), which provide a more comprehensive assessment by incorporating objective measures.

### Collection of clinical data, inclusion and exclusion criteria

Medical records of all consecutive patients who received BEL within a managed-access program after 2–5 prior lines of therapy (LOT) due to severe or therapy-refractory systemic cGVHD were screened. Ocular GVHD was managed with topical treatments and was not classified as therapy‑refractory, as standardized criteria for ocular refractoriness are lacking. Epidemiological data including NIH and ICCGVHD Grading for chronic oGVHD were collected from the patient file. Ophthalmological examinations of the right (OD) and left (OS) eye prior to therapy with BEL and after 3 months included best spectacle-corrected visual acuity (BSCVA), slit lamp bio-microscopy as well as Schirmer’s Test I, corneal esthesiometry (Cochet-Bonnet, Luneau Technology, 2 rue Roger Bonnet, 27340 Pont de l’Arche, France), tear-film-break-up-time (TFBUT), intraocular pressure (IOP) and grading of corneal and conjunctival staining with the Oxford scheme. Subjective symptoms were assessed with a symptom-oriented questionnaire (“Ocular Surface Disease Index”, OSDI®). Exclusion criteria were lack of ophthalmological consultations prior or after establishment of BEL, discontinuation of BEL or missing data.

### Statistical analysis

Descriptive data were collected and analysed by SPSS (version 28.0 for windows; SPSS, Inc, Chicago, IL). The BSCVA was converted to the logarithm of Minimum Angle of Resolution (logMAR). Depending on normal distribution of the interval-scaled parameters we had analysed, we conducted paired t-test and Wilcoxon signed-rank test for statistical analysis. Significant results were defined when *p*-value was <0.05.

## Results

Out of 24 patients, a total of 13 patients (mean age 54.3 ± 14.2) qualified for analysis, as 11 patients were excluded due to lack of ophthalmological consultations prior or after establishment of BEL (*n* = 7), discontinuation of BEL (*n* = 2) or missing data (*n* = 2). For all patients included peripheral blood served as the stem cell source. One patient discontinued BEL due to relapse of leukaemia; the other patient discontinued after 4 weeks due to severe nausea. Demographic variables and patient characteristics are presented in Table 1. Mean number of prior LOTs was 3.7 ± 1.0, including ruxolitinib (RUX) in every patient. Concomitant treatment included RUX in 6 (46.2%) patients, corticosteroids (prednisone 5 mg) in 1 (7.7%) and extracorporeal photopheresis alone (*n* = 1, 7.7%) and with RUX in 1 (7.7%) patient. Time from cGVHD diagnosis to start of BEL was 39.46 ± 38.04 months and time from chronic oGVHD diagnosis was 42.54 ± 35.10 months. Progressive disease (PD) was evident in 5 (38.5%) patients prior to BEL. Overall cGVHD severity was mostly severe (*n* = 11, 84.6%) and in 2 (15.4%) patients graded as moderate. Response rate over all other organs was partial response in 8 patients (61.5%), stable disease in 4 patients (30.8%) and progressive disease in one patient (7.7%).

**Table 1:** Epidemiological and ophthalmological data.

Patient characteristics	Total			
**Demographic variable**	*n* (%)			
	13 (100.0)			
**Gender**				
Male	9 (69.2)			
Female	4 (30.8)			
Mean Age (±SD) in years	54.3 ± 14.2			
**Primary Disease**				
Acute lymphoblastic leukemia (ALL)	2 (15.4)			
Acute myelogenous leukemia (AML)	8 (61.5)			
secondary acute myelogenous leukemia (sAML)	1 (7.7)			
Chronic myelogenous leukemia (CML)	1 (7.7)			
Myelodysplastic syndrome (MDS)	1 (7.7)			
**Donor type**				
matched related donor (MRD)	3 (23.1)			
matched unrelated donor (MUD)	10 (76.9)			
**Conditioning regimen**				
Fludarabine, Busulfan	1 (7.7)			
Fludarabine, Treosulfan	1 (7.7)			
Fludarabin, Treosulfan, Melphalan	2 (15.4)			
Fludarabin, Melphalan	1 (7.7)			
Fludarabin, TBI 8 Gy	1 (7.7)			
FLAMSA, Treosulfan	6 (46.2)			
Cyclophosphamid, TBI 12 Gy	1 (7.7)			
**GVHD prophylaxis**				
CSA, MMF	4 (30.8)			
CSA, MMF, ATG	5 (38.5)			
PT-Cy, MMF, CSA	2 (15.4)			
Tacrolimus, MMF, ATG	1 (7.7)			
PT-Cy, Tacrolimus, MMF	1 (7.7)			
**CMV positive**				
donor	9 (69.2)			
recipient	8 (61.5)			
**Organ involvement cGVHD**				
No. of organs involved, median (range)	3 (2–5)			
≥4 organs involved	5 (38.5)			
skin	13 (100.0)			
joints/fascia	6 (46.2)			
eyes	13 (100.0)			
lungs	4 (30.8)			
liver	8 (61.5)			
**Ophthalmological data**	**prior BEL**	**3 months BEL**		
n=13 (100.0)	**mean **± **SD**	**mean **± **SD**	**95% confidence interval**	***p***-**value**
**ICCGVHD Score**	9.3 ± 2.0	7.5 ± 1.9	[1.27, 2.27]	**0.001**
**BSCVA (logMAR)**				
OD	0.25 ± 0.26	0.18 ± 0.27	[−0.015, 1.092]	0.056
OS	0.15 ± 0.10	0.12 ± 0.11	[−0.175, 0.882]	0.190
**IOP (mmHg)**				
OD	14.31 ± 3.25	13.46 ± 1.90	[−0.273, 0.764]	0.353
OS	14.08 ± 2.84	14.38 ± 2.57	[−0.586, 0.433]	0.768
**Schirmer I (mm)**				
OD	2.31 ± 3.68	3.62 ± 7.1	[−0.708, 0.321]	0.461
OS	1.85 ± 2.30	2.31 ± 3.79	[−0.674, 0.352]	0.539
**Corneal Staining (Oxford)**				
OD	2.31 ± 1.321	1.62 ± 1.19	[0.015, 1.133]	**0.044**
OS	2.31 ± 1.321	1.69 ± 1.03	[−0.091, 0.988]	0.104
**Conjunctival Staining (Oxford)**				
OD	1.85 ± 0.90	1.23 ± 0.60	[0.028, 1.151]	**0.040**
OS	1.85 ± 1.07	1.15 ± 0.69	[0.051, 1.184]	0.032
**Corneal Esthesiometry (cm)**				
OD	5.89 ± 0.33	5.33 ± 1.72	[−0.323, 0.890]	0.360
OS	5.44 ± 0.88	5.50 ± 0.67	[−0.757, 0.433]	0.594
**TFBUT (s)**				
OD	1.73 ± 1.79	2.75 ± 2.73	[−1.093, 0.126]	0.121
OS	1.91 ± 2.12	2.58 ± 2.57	[−0.971, 0.215]	0.213
**OSDI®**	53.84 ± 30.33	51.38 ± 26.72	[−0.405, 0.616]	0.685

### Ophthalmological assessment

ICCGVHD grading before BEL defined 4 (30.8%) patients as mild-moderate and 9 (61.5%) as severe. After 3 months of BEL, 8 (61.5%) of patients were defined as mild-moderate and 5 (38.5%) patients as severe in ICCGVHD grading. There were no changes in NIH grading before and after BEL (prior BEL): NIH grade II: *n* = 3 (23.1%), grade III: *n* = 10 (76.9%). Ophthalmological findings and ICCGHVD scoring prior to starting BEL and after 3 months of BEL treatment are shown in Table 1. Topical steroids were part of the maintenance therapy in 10 patients (76.9%) and topical cyclosporine in 6 patients (46.2%). Allogeneic serum eye drops (SED) were already established in 5 (38.5%) patients and autologous SED in 3 (23.1%) patients. One patient started autologous serum eye drops (SED) during treatment with BEL. However, the patient used autologous finger‑prick blood previously. There are statistically significant differences for ICCGVHD score (*p* = 0.001) and fluorescein staining (cornea: OD *p* = 0.044, conjunctiva: OD *p* = 0.040).

## Discussion

While corticosteroids remain first‑line treatment for cGVHD, up to 50% of patients develop steroid‑refractory or steroid‑dependent disease [8, 9], necessitating more targeted systemic approaches. Evidence comparing immunosuppressive agents for specific organ manifestations remains limited [6, 10, 11], and the reversibility of certain complications—such as severe chronic oGVHD, continues to pose a clinical challenge. In this cohort, treatment with BEL demonstrated clinical efficacy, particularly in reducing ocular surface inflammation and improving ICCGVHD grading. As the ocular surface undergoes relatively rapid epithelial regeneration compared to other organs, early changes may appear unilaterally.

BEL’s antifibrotic effects have been documented in organs such as the skin and lungs, but its impact on ocular fibrosis remains unclear. Schirmer’s test showed no improvement, suggesting that if BEL reduces lacrimal gland fibrosis, it may not reverse acinar cell loss. This aligns with the known cGVHD pathophysiology of the lacrimal gland, where fibrosis represents a currently irreversible consequence of oGVHD [12]. Future studies with fibrosis‑specific measures and earlier intervention may clarify BEL’s potential antifibrotic role in oGVHD.

Symptom response was limited as OSDI® scores did not significantly improve, reflecting advanced disease where irreversible damage could constrain symptomatic relief. The OSDI® itself may undercapture the complexity of chronic oGVHD symptoms, particularly at already high baseline levels. All patients adhered to long‑term topical therapies, which had often reached a therapeutic ceiling prior to BEL initiation. Improvements occurred after BEL initiation, supporting a systemic effect rather than topical adjustments alone. While this therapeutic heterogeneity reflects real‑world clinical practice in chronic oGVHD, it inevitably introduces variability that could influence treatment response dynamics.

All patients had prior ruxolitinib (RUX) treatment and more than half continued RUX, corticosteroids or ECP concomitantly. Despite this, ocular improvements were observed, suggesting BEL may provide additive benefit even in late or refractory disease.

This study is limited by its retrospective design, small sample size, and short follow‑up. Nonetheless, this first real‑world data suggests, that BEL can positively impact chronic oGVHD. Prospective studies with larger cohorts and longer follow‑up are needed to confirm durability of response and elucidate BEL’s role in preventing irreversible damage.

## Data Availability

The datasets generated during and/or analysed during the current study are not publicly available due security and ongoing research.
